# Ruptured Renal Artery Aneurysm in a Pregnant Woman: Case Report and Literature Review

**DOI:** 10.1055/s-0038-1676057

**Published:** 2018-12-12

**Authors:** Adriane Castro de Souza, Caio Henrique Yoshikatsu Ueda, Denise Hiromi Matsubara, João Raphael Zanlorensi Glir

**Affiliations:** 1 Department of Obstetrics and Gynecology, Hospital Santa Cruz, Curitiba, PR, Brazil; 2 Universidade Positivo, Curitiba, PR, Brazil

**Keywords:** renal artery aneurysm, aneurysm in pregnant women, broken aneurysm, gestation, renal aneurysm rupture, aneurisma de artéria renal, aneurisma nas gestantes, aneurisma roto, gestação, ruptura de aneurisma renal

## Abstract

Renal artery aneurysms (RAAs) are rare and usually asymptomatic; ∼ 90% of them are unilateral. Once diagnosed during pregnancy, they may rupture, presenting a high maternal-fetal risk. The present study reports the case of a 32-year-old pregnant woman with a 30-week gestational age and a ruptured unilateral RAA.

## Resumo

Os aneurismas de artéria renal (AAR) são raros, normalmente assintomáticos, e ∼ 90% dos casos são unilaterais. Uma vez diagnosticados durante a gestação, estes podem se tornar predisponentes a rotura e apresentar elevado risco materno-fetal. O presente artigo relata o caso de uma gestante de 32 anos, com idade gestacional de 30 semanas e quadro de AAR unilateral roto.

## Introduction

Renal artery aneurysm (RAA) is a rare and asymptomatic condition in the general population and, although some risk factors can be recognized, its etiology is still controversial. The most accepted etiology is that the disease results from the loss of elastic fibers and from the decrease of the smooth muscle tissue of the middle layer.^1^ Pregnancy has characteristics that may contribute to the increased size and rupture of RAAs, such as the hyperdynamic state and increased intra-abdominal pressure.^2^

The rupture of RAAs may cause symptoms that can be confused with other common conditions during pregnancy. It is an emergency and a complication that has an unfavorable prognosis for both the pregnant woman and the fetus. The present study reports the case of a pregnant woman who presented to the emergency room with symptoms that initially suggested pyelonephritis. However, as the case evolved, the patient was diagnosed with a ruptured renal artery.

## Case Description

A 32-year-old female, previously healthy, with history of 1 previous caesarian section and 30 weeks pregnant at admittance, presented to the emergency room with lower back and abdominal pain associated with vomiting that had begun 1 hour before. At admittance, the vital signs were: blood pressure of 90/45 mm Hg, temperature of 36.2° C, and oxygen saturation of 98%. During the initial examination, pain upon palpation of the iliac fossa and hypogastrium was observed, with no signs of peritoneal irritation, and positive Giordano test on the left side of the back. Furthermore, during the obstetric gynecological examination, it was observed that the fetal heart rate was present and that the cervix was thick, long, posterior, and impervious. Due to the initial hypothesis of pyelonephritis associated with septic shock, blood culture and laboratory tests were performed. An obstetric ultrasonography (USG) and a room in the intensive care unit (ICU) were also requested, and ceftriaxone and fluid stat were initiated.

**Fig. 1:**
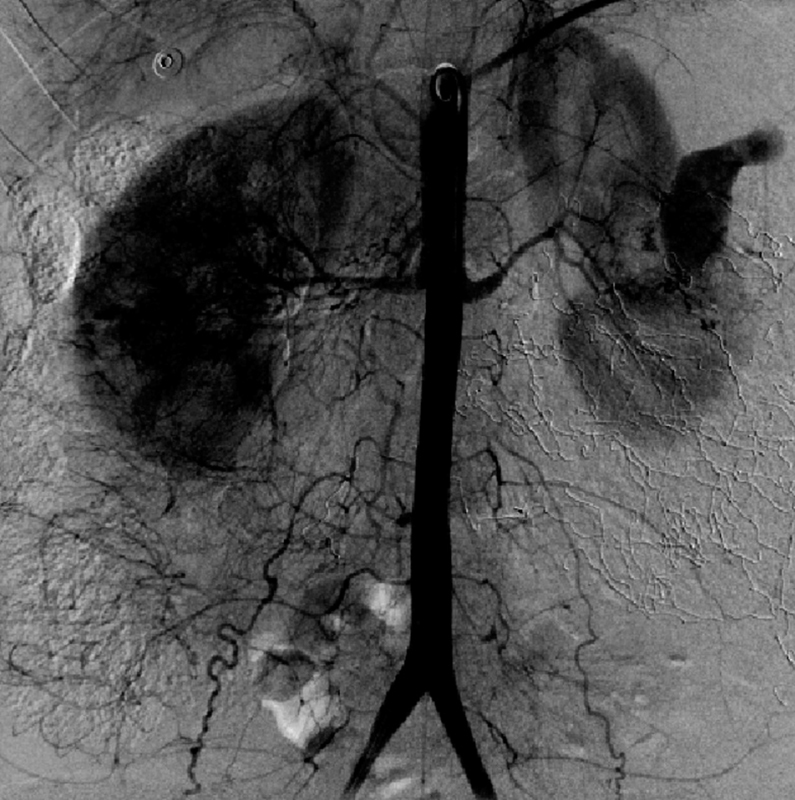
Arteriography demonstrating extravasation of contrast by the ruptured aneurysm.

As a result of the worsening of the hemodynamic state, the patient was transferred to the ICU 2 hours after admittance. At that time, the obstetric USG exam demonstrated the absence of fetal movements and cardiac activity, in addition to a large amount of thick fluid in the abdominal cavity, suggestive of hemoperitoneum, which was subsequently confirmed by an abdominal USG. A red blood cells transfusion and an emergency cesarean section were then performed, the latter of which confirmed fetal death and hemorrhage, resulting in a large amount of blood in the retroperitoneum. The general surgeon opted for damage control with retroperitoneal tamponade and a reapproach within between 48 and 72 hours due to the hemodynamic instability. Owing to the critical situation, it was necessary to use vasoactive drugs (VADs), orotracheal intubation (OTI), and sedation (Fig. 1).

Once the patient was stable, an emergency arteriography showed contrast extravasation by the left renal peripheral branch located in the inferolateral region (Fig. 1), and a super selective catheterization with embolization was requested. However, an abdominal USG exam evidenced a large amount of blood in the abdominal cavity, and owing to the risk of Disseminated intravascular coagulation (DIC), a new approach to cavity lavage was discussed with the general surgeon. In this approach, there was the presence of blood in the abdominal cavity that culminated in a new retroperitoneal tamponade and in a reapproach in between 48 and 72 hours. The patient remained in ICU care, still with VAD and OTI, for 2 days, when a new approach was taken; an ischemic lesion in the sigmoid was found, thus requiring sigmoidectomy. During the following days, there were adjustments to the antibiotic therapy, to the VAD, and to the sedoanalgesia, with progressive improvement of the condition of the patient. After the 7^th^ day in the ICU, the patient was discharged and was referred the hospital ward. On the 3^rd^ day after the discharge from the ICU, the condition of the patient evolved with a large amount of fecal discharge through the surgical drain, which was associated with tachycardia, tachypnea, and hypoxemia. The patient was referred back to the ICU with secondary peritonitis due to a probable enteric fistula. New imaging tests were requested, which showed pleural effusion and a subphrenic abscess on the right region, pneumoperitoneum, and sigmoid colon fistula. Owing to this complication, a new abdominal approach was required for surgical drainage of the abdominal abscess, as well as a thoracoscopy for chest drainage. The patient remained in the ICU for 15 days to control the abdominal infection and was stable and without new organ dysfunctions. The patient was then transferred to the hospital ward so that her nutritional status could be monitored and for the control and correction of the fistula. After 15 days, the patient showed clinical improvement and was then discharged.

## Discussion

Renal artery aneurysms are defined as a localized dilation of the renal artery or of its branches.^3^ It is a rare pathology with an incidence close to 0.1% in the general population, and the rupture of these aneurysms is even less frequent, occurring in ∼ 2% of this population.^4^ ^5^ However, the prognosis of a ruptured RAA is not favorable, mainly for pregnant women, with maternal death rates varying between 50 and 92%, and fetal death rates ranging between 82 and 100%.^6^

The most common RAA etiology is that the disease results from the loss of elastic fibers and from the decrease of the smooth muscle tissue of the middle layer^1^ The risk factors include systemic arterial hypertension (SAH), atherosclerosis, fibromuscular dysplasias, changes in collagen metabolism, inflammatory diseases, penetrating or blunt trauma, female gender, being ≥ 60 years old, and pregnancy.^3^ ^4^ It is believed that the increase of plasma volume, intra-abdominal pressure, hormonal-metabolic changes, and hyperdynamic state in pregnancy may be contributing factors not only for the increased size but also for the rupture of these aneurysms. However, there seems to be no relation between the number of pregnancies and RAAs.^2^

Most patients have saccular and asymptomatic aneurysms. Pain, hypertension, and hematuria may be observed in symptomatic cases. Symptoms like murmur and palpable abdominal mass are non-specific and unreliable signs during pregnancy. The main complication of RAA is its rupture, and in such cases, the patient may have sudden and non-specific abdominal or lumbar pain, usually in the costovertebral angle, hypovolemic shock, gross hematuria, or complications owing to the risk of embolization of thrombi arising from the aneurysm. This makes it difficult to distinguish it clinically from other conditions, such as the causes of acute abdomen aetiology (infection, inflammation, vascular occlusion or obstruction), pyelonephritis, or ectopic pregnancy.^4^ ^7^ ^8^

As most RAAs are asymptomatic, the diagnosis is usually made incidentally through imaging tests. Angiography is considered the gold standard, owing to its diagnostic capacity, preoperative analysis, and the possibility of immediate correction through embolization. Otherwise, a computed tomography (CT) exam can be performed because it is a sensitive examination, and, in an emergency case, the benefits are worth the risk. Ultrasonography is an examination that does not pose maternal-fetal risks and can be performed in hemodynamically unstable patients; however, it does not always identify the aneurysm. Magnetic resonance imaging (MRI) is not recommended in cases of instability. Laparoscopy may have its diagnostic value compromised in pregnant women, mainly after the second trimester, when the size of the uterus may be a limiting factor for the method.^3^ ^5^

The treatment for pregnant women with RAA is quite controversial in the literature, and the conduct depends on the following factors: size and location of the aneurysm, presence or absence of symptoms, and the existence of renovascular hypertension and calcifications.^6^

To quantify the risk of rupture of these aneurysms, González et al^9^ suggest that RAAs pose risks ranging from low risk of rupture (calcified aneurysms with a diameter of < 1.5 cm) to those with > 20% chance of rupture (non-calcified saccular aneurysms associated with SAH). Thus, RAAs, in general, should be treated in the following situations: when the lesions measure > 2 cm in diameter, when they show documented growth, when they are symptomatic, before documented distal embolization, when they are associated with significant stenosis and poor renal perfusion, and in women of childbearing age with a desire of future pregnancies or pregnant women.^9^ ^10^

The conduct in the case of a ruptured renal aneurysm in a pregnant woman is based primarily on clinical suspicion and on a rapid diagnosis with the control of hemodynamic instability. An emergency cesarean section should be performed to avoid fetal death, followed by a surgical approach for hemorrhage control. Subsequently, an endovascular correction with embolization can be performed or, in situations where there is no hemodynamic services available, nephrectomy may be selected.

## Final Considerations

Although rare, RAAs may evolve unfavorably, especially in pregnancy. The present case report demonstrates the complexity and difficulty associated with the clinical assessment of ruptured RAAs and their complications, even in assisted patients. Therefore, a fast and correct diagnosis, associated with assessment techniques and commitment of the medical team are fundamental so that the morbimortality of these cases is as low as possible.
